# Serum amyloid A expression in the breast cancer tissue is associated with poor prognosis

**DOI:** 10.18632/oncotarget.8561

**Published:** 2016-04-02

**Authors:** Mu Yang, Fangfang Liu, Kayoko Higuchi, Jinko Sawashita, Xiaoying Fu, Li Zhang, Lanjing Zhang, Li Fu, Zhongsheng Tong, Keiichi Higuchi

**Affiliations:** ^1^ Department of Aging Biology, Institute of Pathogenesis and Disease Prevention, Shinshu University Graduate School of Medicine, Matsumoto, Japan; ^2^ Department of Breast Pathology and Research Laboratory, Key Laboratory of Breast Cancer Prevention and Therapy (Ministry of Education), National Clinical Research Center for Cancer, Tianjin Medical University Cancer Institute and Hospital, Tianjin, China; ^3^ Department of Pathology, Aizawa Hospital, Matsumoto, Japan; ^4^ Department of Biological Sciences for Intractable Neurological Diseases, Institute for Biomedical Sciences, Interdisciplinary Cluster for Cutting Edge Research, Shinshu University, Matsumoto, Japan; ^5^ Department of Pathology, Tianjin University of Traditional Chinese Medicine, Tianjin, China; ^6^ Department of Breast Oncology, Key Laboratory of Breast Cancer Prevention and Therapy (Ministry of Education), National Clinical Research Center for Cancer, Tianjin Medical University Cancer Institute and Hospital, Tianjin, China; ^7^ Department of Pathology, University Medical Center of Princeton, Plainsboro, NJ, USA; ^8^ Cancer Institute of New Jersey, New Brunswick, NJ, USA; ^9^ Department of Pathology, Robert Wood Johnson Medical School, Rutgers University, New Brunswick, NJ, USA; ^10^ Department of Chemical Biology, Ernest Mario School of Pharmacy, Rutgers University, Piscataway, NJ, USA

**Keywords:** breast carcinoma, serum amyloid A, tumor marker, survival, macrophages

## Abstract

**Background:**

Serum amyloid A (SAA), an acute-phase protein, is expressed primarily in the liver, and recently found also expressed in cancer tissues. However, its expression and prognostic value in breast cancer have not been described.

**Results:**

SAA protein was found expressed in tumor cells in 44.2% cases and in TAM in 62.5% cases. FISH showed more frequent SAA mRNA expression in TAM than in tumor cells (76% versus 12%, *p* < 0.001), and a significant association between the frequencies of SAA mRNA expression in TAM and tumor cells (*r*_s_ = 0.603, *p* < 0.001). The immunoreactivities of SAA protein in TAM and tumor cells were both associated with lymphovascular invasion and lymph node metastasis. Moreover, SAA-positivity in TAMs was associated with larger tumor-size, higher histological-grade, negative estrogen-receptor and progesterone-receptor statuses, and HER-2 overexpression. It was also linked to worse recurrence-free survival in a multivariable regression model.

**Methods:**

Immunohistochemistry was applied on the tumor tissues from 208 breast cancer patients to evaluate the local SAA-protein expression with additional CD68 stain to identify the tumor-associated macrophage (TAM) on the serial tissue sections. Fluorescent *in situ* hybridization (FISH) was conducted on serial tissue sections from 25 of the 208 tumors to examine the expression and location of SAA mRNA.

**Conclusions:**

Our results suggested that the TAMs may be a pivotal and main source of SAA production in tumor microenvironment of breast cancer. SAA immunoreactivity in TAM is associated with worse recurrence-free survival, and is therefore a biomarker candidate for postoperative surveillance and perhaps a therapeutic target for breast cancer.

## INTRODUCTION

Serum amyloid A (SAA), a positive acute-phase protein, is generated primarily by the liver in response to trauma, infection, inflammation, and even neoplastic stimuli. There are four SAA isotypes in humans [1]. SAA1 and SAA2 each consist of 122 amino acids, including signal peptide sequences, and share more than 90% of their amino acid sequences [2]. In addition, SAA3 is a pseudogene, and SAA4 is constitutively expressed at a constant level and is thus known as cSAA. On the other hand, the production of SAA1 and SAA2 by hepatocytes is 100- to 1000-fold upregulated during the acute phase response. In physiological conditions, SAA is present in various forms, including SAA1 and SAA2 [3]. Distinct isoforms of SAA1 and SAA2 exist (SAA1α, SAA1β and SAA1γ and SAA2α and SAA2β), and differ only in a few amino acids [4, 5]. In our study, we used a polyclonal antibody for immunohistochemistry (IHC) and an isoform primer for fluorescent *in-situ* hybridization (FISH) to detect the protein and mRNA of both SAA1 and SAA2, respectively.

The acute-phase SAAs (aSAA), SAA1 and SAA2, increase in concentration approximately several hundred-fold in response to inflammatory stimuli [6, 7]. Studies suggest that aSAA could considerably influence carcinogenesis by activating the transcriptional factor nuclear factor kappa-B (NFκB) [8–12] and inducing the expression of matrix metalloproteinases (MMPs) [13–15]. The activation of these genes could suppress apoptosis [16]. Recent studies have revealed other sources of SAA outside of the liver: specifically, the cancer tissues of the esophagus, lung, pancreas, ovary, uterine endometrium and uterine cervical cancer [17–22]. However, the association of its expression in different distributions of tumor tissue with clinicopathological features and prognosis in cancer tissues has not been well described.

To the best of our knowledge, the localized protein and mRNA expression of SAA in breast cancer tissue has not yet been reported. In this study, using IHC and FISH, we aimed to examine the SAA expression levels and locations in breast cancer tissues, and their potential association with the overall and recurrence-free survivals, and clinicopathological features, according to REMARK recommendations [23].

## RESULTS

### SAA expression by IHC and FISH in breast cancer

**Figure 1:**
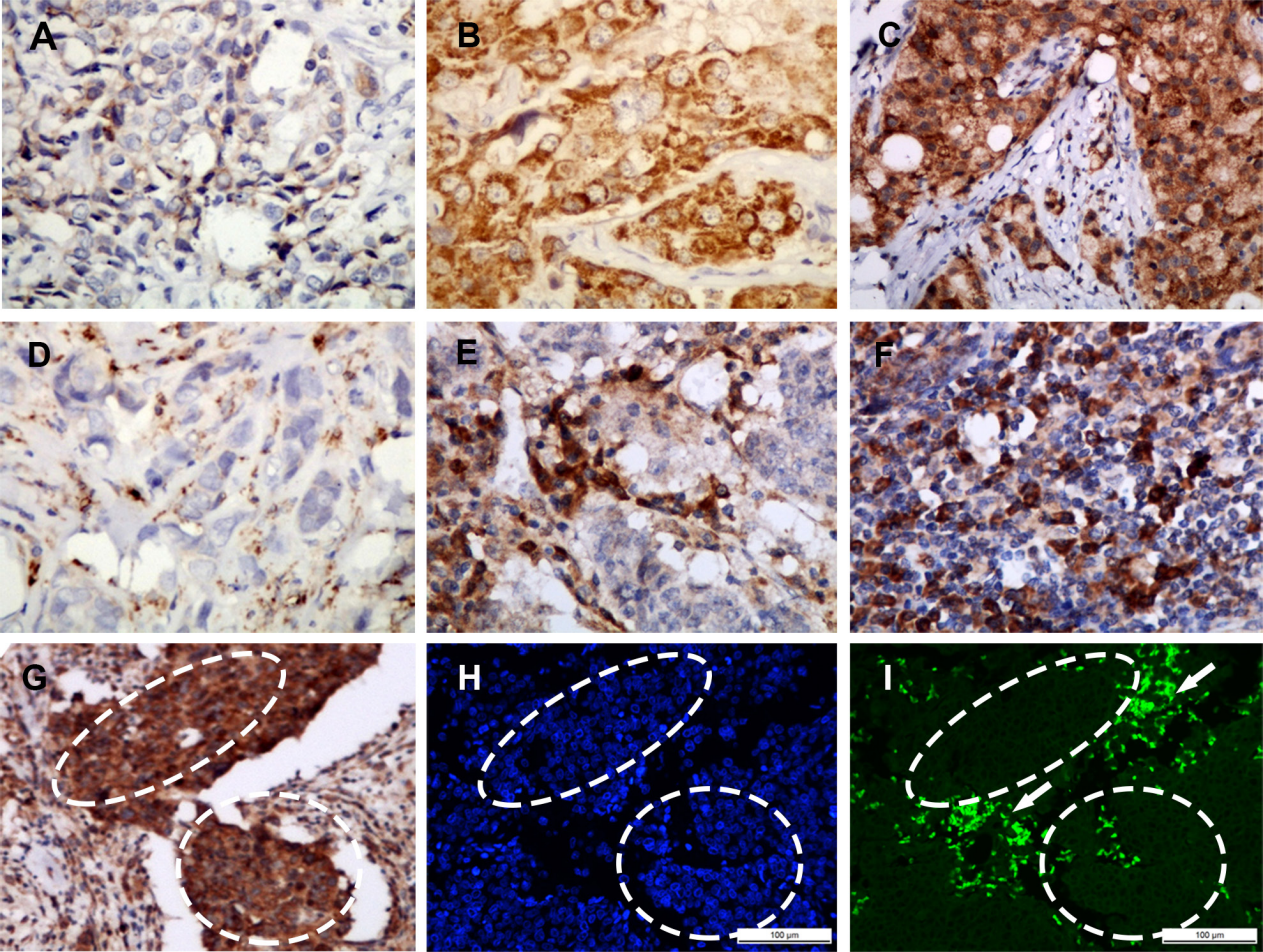
Representative SAA staining intensities by immunohistochemistry Staining was localized to the cytoplasm or to the membrane of tumor cells (**A–C**): a, Intensity 1+, no or weak staining; b, Intensity 2+, moderate staining; c, Intensity 3+, strong staining. Representative infiltration densities of SAA+ macrophage by immunohistochemistry (**D–F**): d, 1+, sparse; e and f, 2+, median to dense. SAA protein and mRNA were identified by immunohistochemistry (**G**) and *in situ* hybridization (**H** and **I**), respectively, on serial paraffin tissue sections. The local over-expression of SAA at the protein level was demonstrated mainly in tumor cell (inside the dashed area) and stromal infiltrated macrophages (outside the dashed area) (G), but the SAA mRNA was mainly located in the macrophages (outside the dashed area, shown by the arrow) and rarely in the tumor cells (inside the dashed area) SAA mRNA = Green (I) Cell nucleus = Blue (H) (H and I). A–I, magnification × 200.

Among the 208 invasive breast cancer samples, SAA protein was found expressed in tumor cell in 44.2% (92/208) cases (Figure 1A–1C) and expressed in macrophage in 62.5% (130/208) cases (Figure 1D–1F). A positive correlation with a high correlation coefficient was found between SAA protein expression levels in tumor cells and macrophages (*r*_s_ = 0.603, *p* < 0.001) (Table 1).

**Table 1:** Patient information and clinicopathologic parameter correlations with SAA expression

	SAA in tumor cell				SAA in macrophage				
Characteristics	− (%)	+ (%)	*r_s_*	*P*	− (%)	+ (%)	++ (%)	*r_s_*	*P*
Patient number	116 (55.8)	92 (44.2)			78 (37.5)	74 (35.6)	56 (26.9)		
Age, years									
Median (range)	56 (27–89)	55 (30–80)	−0.012	0.864	56 (29–79)	55 (27–89)	56 (30–75)	−0.016	0.815
Tumor size, cm									
Median (range)	2.3 (0.8–11)	2.3 (0.2–7.5)	0.002	0.979	2.1 (0.8–4)	2.2 (0.2–9)	2.7 (0.9–11)	0.225	0.001
No. of Lymph nodes involved									
Mean ± SD	2.2 ± 5.6	2.8 ± 5.8	0.191	0.006	1.2 ± 3.8	2.1 ± 4.7	4.8 ± 8.0	0.387	< 0.001
range	0–32	0–38			0–23	0–32	0–38		
Lymph node stage									
N0	78 (65.5)	41 (34.5)	0.198	0.004	62 (52.1)	42 (35.3)	15 (12.6)	0.401	< 0.001
N1	20 (39.2)	31 (60.8)			8 (15.7)	20 (39.2)	23 (45.1)		
N2	8 (38.1)	13 (61.9)			5 (23.8)	7 (33.3)	9 (42.9)		
N3	10 (58.8)	7 (41.2)			3 (17.6)	5 (29.4)	9 (52.9)		
Definite LVI									
Negative	52 (68.4)	24 (31.6)	0.193	0.005	41 (53.9)	25 (32.9)	10 (13.2)	0.288	< 0.001
Positive	64 (48.5)	68 (51.5)			37 (28)	49 (37.1)	46 (34.9)		
Nuclear grade									
I	13 (76.5)	4 (23.5)	0.062	0.372	13 (76.5)	3 (17.6)	1 (5.9)	0.202	0.003
II	88 (53.3)	77 (46.7)			59 (35.8)	59 (35.8)	47 (28.5)		
III	15 (57.7)	11 (42.3)			6 (23.1)	12 (46.2)	8 (30.8)		
ER									
Negative	19 (46.3)	22 (53.7)	−0.094	0.177	5 (12.2)	19 (46.3)	17(41.5)	−0.252	< 0.001
Positive	97 (58.1)	70 (41.9)			73 (43.7)	55 (32.9)	39(23.4)		
PR									
Negative	25 (46.3)	29 (53.7)	−0.113	0.104	8 (14.8)	26 (48.1)	20 (37)	−0.250	< 0.001
Positive	91 (59.1)	63 (40.9)			70 (45.4)	48 (31.2)	36 (23.4)		
HER-2 status									
Negative	89 (58.2)	64 (41.8)	0.081	0.247	66 (43.1)	52 (34)	35 (22.9)	0.204	0.003
Positive	27 (49.1)	28 (50.9)			12 (21.8)	22 (40)	21 (38.2)		
CD68									
−	10 (76.9)	3 (23.1)	0.200	0.004	13 (100)	0 (0)	0 (0)	0.690	< 0.001
+	66 (62.3)	40 (37.7)			60 (56.6)	41 (38.7)	5 (4.7)		
++	40 (44.9)	49 (55.1)			5 (5.6)	33 (37.1)	51 (57.3)		
SAA in tumor cell									
Low					69 (59.5)	40 (34.5)	7 (6.0)	0.603	< 0.001
High					9 (9.8)	34 (37.0)	49 (53.3)		

Note: SAA: serum amyloid A; LVI: lymphovascular invasion; Lymph node stage: N0, indicates no lymph node metastasis; N1, 1–3 lymph node metastasis; N2, 4–9 lymph node metastasis; N3, ≥ 10 lymph node metastasis; ER: estrogen receptor; PR: progesterone receptor; HER-2: human epidermal growth factor receptor-2.

FISH showed that SAA mRNA was predominantly located in tumor-associated macrophage (TAM) (76%, 19/25), and was also detected in some infiltrating lymphocytes. In contrast, SAA mRNA was rarely identified in the tumor cells (12%, 3/25), even in the cases with SAA immunopositivity in the tumor cells (13%, 2/15) (Table 2). The SAA mRNA expression rate (76%, 19/25 versus 12%, 3/25; *p* < 0.001) and intensity in macrophages were significantly higher than that in tumor epithelial cells (Figure 1G–1I). In addition, SAA mRNA expression was found positively correlated with protein expression in TAM (*r*_s_ = 0.508, *p* < 0.010) but not in tumor cells (*r*_s_ = 0.050, *p* = 0.811) (Table 2).

**Table 2:** SAA expression by IHC and FISH in breast cancer

IHC	FISH		*r_s_*	*P* values
	−	+		
Tumor cells				
−	9	1	0.050	0.811
+	13	2		
Macrophages				
−	3	0	0.508	0.010
+	2	8		
++	1	11		

Note: SAA: serum amyloid A; IHC: immunohistochemistry; FISH: Fluorescent *in situ* hybridization.

### SAA expression is correlated with some clinicopathological parameters of breast cancer patients

The SAA immunoreactivities in tumor cells and macrophage were both positively associated with lymphovascular invasion (*r*_s_ = 0.193, *p* = 0.005; *r*_s_ = 0.228, *p* < 0.001, respectively), higher lymph node stage (*r*_s_ = 0.198, *p* = 0.004; *r*_s_ = 0.401, *p* < 0.001, respectively), and more lymph nodes with tumor metastasis (*r*_s_ =0.191, *p* = 0.006; *r*_s_ = 0.387, *p* < 0.001, respectively). Moreover, SAA positive macrophages were also associated with larger tumor size (*r*_s_ = 0.225, *p* = 0.001), higher histological grade (*r*_s_ = 0.202, *p* = 0.003), negative estrogen-receptor (*r*_s_ = −0.252, *p* < 0.001) and progesterone-receptor statuses (*r*_s_ = −0.250, *p* < 0.001), and human epidermal growth factor receptor 2 (HER- 2) overexpression (*r*_s_ = 0.204, *p* = 0.003) (Table 1). SAA protein expressions in tumor cells and in macrophage were both correlated with CD68^+^ macrophage infiltration in tumor tissue.

### Prognostic significance of SAA expression

**Figure 2:**
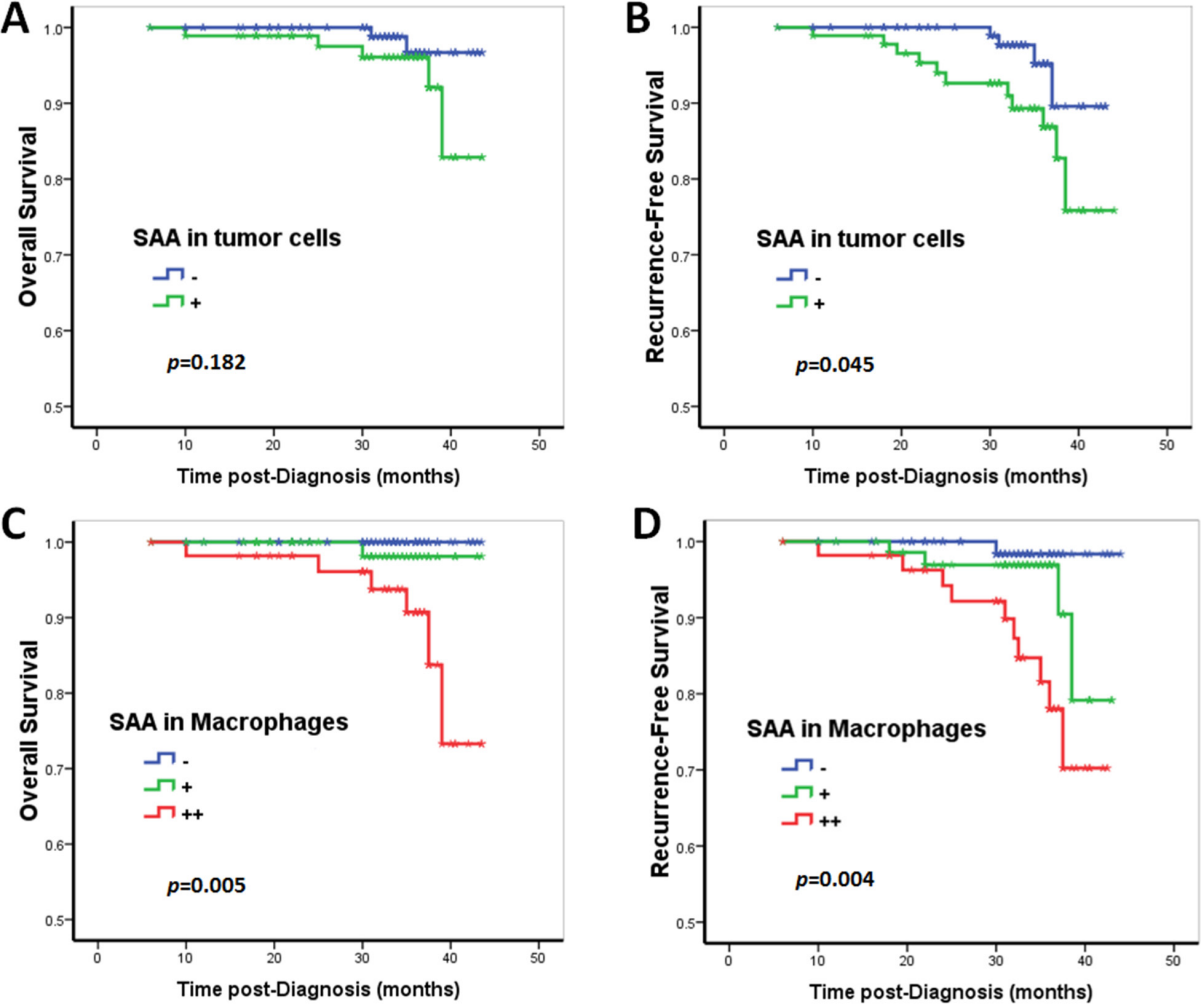
Prognostic significance of SAA expression in breast cancer patients Kaplan–Meier survival curve for (**A**, **C**) overall survival (OS) and (**B**, **D**) recurrence-free survival (RFS) depending on the expression of SAA. Patients with SAA expression in tumor cells showed a decreased RFS (B). Although patients with tumor cell SAA expression showed a relative decreased OS, the results did not reach statistical significance (A). SAA+ macrophages were an unfavorable predictor for OS and RFS (C and D). In contrast, *p*-values were calculated by the log-rank test.

The univariate Cox proportional hazards regression analysis confirmed that SAA immunoreactivity in TAM was an unfavorable predictor for overall survival (OS, HR [hazard ratio] = 8.73, *p* = 0.027) and recurrence-free survival (RFS: HR = 3.34, *p* = 0.003) (Tables 3 and 4; Figure 2). In contrast, although SAA immunoreactivity in tumor cells predicted a worse prognosis, there was no statistical significance in the univariate Cox proportional hazards regression analysis (OS: HR = 2.91, *p* = 0.203; RFS: HR = 3.06, *p* = 0.057) (Tables 3 and 4; Figure 2). In the multivariate Cox proportional hazards regression analysis, after adjusting for age, tumor size, grade, lymph node stage and CD68+ macrophage, SAA immunoreactivity in macrophages was proved to be an independent negative prognostic factor for RFS (HR = 2.33, *p* = 0.046; Table 4), but not for OS (HR = 6.26, *p* = 0.063; Table 3).

**Table 3:** Univariate and multivariate Cox proportional hazards regression analyses on the factors associated with overall survival in breast cancer

Factors	Univariate			Multivariate		
	HR	95% CI	*P* values	HR	95% CI	*P* values
Age	0.94	0.87–1.02	0.144	0.94	0.87–1.02	0.167
Tumor size	1.50	1.13–1.99	0.005	1.23	0.87–1.73	0.237
Lymph node stage (N0 vs. N1 vs. N2 vs. N3)	2.72	1.33–5.54	0.006	2.20	1.04–4.66	0.040
Nuclear grade (1 vs. 2 vs. 3)	2.79	1.13–6.92	0.027	2.25	0.93–5.42	0.071
SAA in tumor cell (− vs. +)	2.91	0.56–15.09	0.203	1.02	0.10–10.32	0.985
SAA^+^ macrophage (− vs. + vs. ++)	8.73	1.28–59.41	0.027	6.26	0.91–43.11	0.063
CD68^+^ macrophage (− vs. + vs. ++)	6.72	0.86–52.59	0.070	1.20	0.12–11.54	0.876

Note: SAA: serum amyloid A; vs.: versus. Lymph node stage: N0, indicates no lymph node metastasis; N1, 1–3 lymph node metastasis; N2, 4–9 lymph node metastasis; N3, ≥ 10 lymph node metastasis.

**Table 4:** Univariate and multivariate Cox proportional hazards regression analyses on the factors associated with recurrent free survival in breast cancer

Factors	Univariate			Multivariate		
	HR	95% CI	*P* values	HR	95% CI	*P* values
Age	0.95	0.90–1.00	0.055	0.94	0.88–1.00	0.061
Tumor size	1.37	1.09–1.72	0.007	1.08	0.82–1.42	0.601
Lymph node stage (N0 vs. N1 vs. N2 vs. N3)	2.64	1.64–4.23	< 0.001	2.31	1.35–3.94	0.002
Nuclear grade (1 vs. 2 vs. 3)	2.06	1.21–3.48	0.007	1.47	0.85–2.54	0.164
SAA in tumor cell (− vs. +)	3.06	0.97–9.67	0.057	1.97	0.66–5.88	0.227
SAA^+^ macrophage (− vs. + vs. ++)	3.34	1.49–7.48	0.003	2.33	1.02–5.33	0.046
CD68^+^ macrophage (− vs. + vs. ++)	3.56	1.21–10.52	0.021	1.12	0.26–4.79	0.882

Note: SAA: serum amyloid A; vs.: versus. Lymph node stage: N0, indicates no lymph node metastasis; N1, 1–3 lymph node metastasis; N2, 4–9 lymph node metastasis; N3, ≥ 10 lymph node metastasis.

## DISCUSSION

### Presence of SAA in breast cancer cells and TAMs

Recent studies reported that cancer tissue might be a major source of SAA outside of the liver, such as in the patients with esophagus, lung, pancreas, ovary, uterine endometrium or uterine cervical cancer [17–22]. In this study, we showed the presence of SAA protein in both tumor cells and macrophages in human breast carcinomas, and significantly more frequent expression of SAA mRNA in macrophages than tumor cells. In addition, a significant correlation was found between the SAA mRNA and protein expression levels in TAM, but not that in tumor cells. These results indicated that TAM rather than tumor cell might be a main source of SAA production in tumor microenvironment.

As we know, endothelial cell (EC) secret a limited number of cytokines and many of these present inside come from other cell sources by transcytosis [24–26]. In present study, we found a positive correlation with a high correlation coefficient between SAA protein expression in tumor cells and surrounding TAMs. Therefore, overexpression of SAA protein in tumor cells may reflect a high level of SAA protein secreted by the TAM in tumor microenvironment of breast cancer. In contrast, many researchers studied the local SAA genes' expression by RT-PCR analysis on RNA extracted from cancer biopsy specimens and speculated that the tumor cells are the main secretion source of SAA in local tumor tissues [21, 22]. One limitation of this method, unless combining tissue microdissection technique, is its inability to identify the location and secretion source in the complex cell components in tumor microenvironment. Our methods and findings seem to overcome this methodological limitation and fill in the knowledge gap on the source of SAA in the breast cancer tissue. However, it is still unclear how the SAA is relocated from TAM to tumor cells and more works are needed.

### SAA immunoreactivity in TAM and breast cancer recurrence

We also show that the elevated SAA level in breast cancer cells is associated with more advanced lymph node stage and lymphovascular invasion in breast cancer patients, while SAA immunoreactivity in TAM associated with almost all the important clinicopathological parameters. More interestingly, SAA immunoreactivity in TAM was proved to be an independent negative prognostic factor for RFS of breast cancer. In contrast, although patients with tumor cell SAA expression showed a relatively worse prognosis, the results did not reach statistical significance. These lines of evidence are also consistent with more frequent mRNA expression of SAA in TAM than tumor cells in breast cancer. Therefore, it appears reasonable to speculate that the TAMs rather than the tumor cells are a pivotal location and a main source of SAA production in tumor microenvironment, and the SAA immunoreactivity of TAM may be a prognostic factor for breast cancer.

It has been documented that SAA chemoattracts monocytes, lymphocytes, and granulocytes [27–29] through binding and signaling via its high-affinity G protein-coupled receptor formyl peptide receptor like 1/formyl peptide receptor 2 (FPRL1/FPR2) [30]. Given that inflammation has recently been proposed to be associated with tumorigenesis [31, 32], SAA may increase local inflammation in the microenvironment of the malignant tissue by inducing the production of pro-inflammatory cytokines, tumor necrosis factor-α (TNFα), interleukin- 1b (IL-1), and the chemokines CCL1, CCL3 and CCL4 [33–35]. As far as we know, SAA gene has been indicated to be expressed in monocyte/macrophage cells in an early report [36]. Our FISH data suggest that the main source of SAA in the local tissue of breast cancer is the TAM surrounding the tumor epithelial cells. Recently, Gouwy M *et al.* [37] reported that SAA1 induced significant amounts of macrophage inflammatory protein-1α/CC chemokine ligand 3 (MIP-1α/CCL3) and interleukin-8/CXC chemokine ligand 8 (IL-8/CXCL8) in monocytes and dendritic cells (DCs) in a dose-dependent manner. SAA1 also directly activated monocytes and DCs for signaling and chemotaxis without chemokine interference. In addition to direct leukocyte activation, SAA1 induces a chemotactic cascade mediated by expression of cooperating chemokines to prolong recruiting leukocytes the inflammatory site. TAMs have been shown to enhance tumor progression by promoting tumor invasion, migration and angiogenesis in several solid tumors [38, 39]. Our results suggest that increased SAA secretion by TAM may also contribute to the tumor progression and metastasis of breast cancer, while more studies are needed to elucidate the related molecular mechanisms.

### Study limitations

Several limitations of this study are noteworthy. First, only a handful of deaths occurred during our follow-up of the 208 patients. The relatively small number of deaths may lead to an insufficient statistical power to identify potentially significant factors, such as the association between SAA immunoreactivity in TAM and OS. Second, our follow-up time of 34 months (median) is reasonably long, but an even longer follow-up might have provided more accurate survival estimation. Caution therefore should be used when interpreting and applying our findings. Taken together, prospective and larger studies with longer survival are needed to validate our findings.

In conclusion, we here show the first evidence of local expression of SAA in human breast cancer, and more frequent SAA mRNA and protein expressions in TAM than tumor cells. This finding suggests that TAM is a main source of SAA in breast cancer microenvironment. We also show that SAA, especially expressed in the TAMs, is associated with worse RFS, and may be a potential biomarker for postoperative surveillance and perhaps a therapeutic target in breast cancer.

## MATERIALS AND METHODS

### Breast cancer patients

We included 208 patients with invasive breast cancer diagnosed at Tianjin Cancer Hospital, Tianjin, China in 2011. The patients were followed up for 6–44 months (median, 34 months), and the range of the patients age at the time of diagnosis was 27–89 years (median, 55 years). During the follow-up, 8 patients (3.8%) died and 16 patients (7.7%) had a breast cancer recurrence. All the patients presented with tumors that were confined to the breast, without evidence of distant metastasis or skin involvement at presentation. All the patients underwent modified radical mastectomy (91.8%, 191/208) or breast-conserving surgery (8.2%, 17/208) with complete axillary lymph node dissection. No patients had received neoadjuvant chemotherapy or preoperative radiation therapy. Postoperatively, 191 (91.8%) patients received adjuvant chemotherapy, 81 (38.9%) received radiation therapy, and 165 (79.3%) received endocrine therapy. All patients signed an informed consent form for participation in the study and for the use of their biological tissues. The study was reviewed and approved by the Institutional Ethics Committee of Tianjin Medical University Cancer Institute and Hospital, Tianjin, China (No.bc2015005). The study protocol was also approved by the Institutional Review Board on Human Experiments, Shinshu University School of Medicine (No.3237).

### Immunohistochemistry

The IHC for SAA and CD68 was performed on serial whole-tissue sections using standard procedures. Briefly, 4-μm tissue sections were cut from the archived formalin fixed paraffin embedded tissue blocks, sequentially dewaxed with xylene and rehydrated with graded alcohol washes. Antigen retrieval was performed at 121°C for 2 min using citrate buffer, pH 6.0. After serial blocking with hydrogen peroxide and normal goat serum, the sections were incubated with a primary polyclonal antibody against SAA (SAA1 and SAA2, Abcam, EPR4134, 1:100 dilution, Cambridge, UK) or CD68 (Abcam, ab955, clone KP1, monoclonal, 1:100 dilution, Cambridge, UK) for 16 h at 4°C. The sections were then sequentially incubated with biotinylated goat anti-mouse immunoglobulin and peroxidase-conjugated streptavidin (DAKO, Denmark). The enzyme substrate was 3, 3′-diaminobenzidine tetra-hydrochloride. Incubation of sections with phosphate-buffered saline alone served as a negative control.

SAA expression in the cell cytoplasm or on the membrane was considered a positive result. The staining intensity and the frequency of SAA expression in tumor cells were assessed using the Histo-score (H-score). The staining of the entire slide was scored by intensity (1+ = weak, 2+ = moderate, 3+ = strong) (Figure 1A–1C) and percentage of invasive tumor cells stained for each intensity. The H-score was calculated using the following formula: (3 × percentage of strong staining) + (2 × percentage of moderate staining) + (1 × percentage of weak staining), with the possible scores ranging from 0 to 300. SAA expression was classified into two groups according to a cut-off H-score of 100 (0–99 = negative/low expression; 100–300 = positive expression).

The CD68 or SAA staining in macrophage was scored according to the infiltration density of CD68^+^ or SAA1^+^ cells with a monocyte/macrophage morphology, ranging from 0 (absent) up to 3 (dense) (Figure 1D–1F). For statistical analyses, these cases were categorized into - (absent, 0), 1+ (sparse, 1) or 2+ (moderate to dense, scores 2–3) macrophage-infiltration groups.

### Fluorescent *in situ* hybridization

FISH was conducted on serial tissue sections from 25 tumors randomly selected from the 208 tumors to examine the SAA mRNA expression and location in tumor tissues. PCR products were generated, and FISH was performed with mRNA-targeted fluorescence labelled oligonucleotide probes. Hybridization probes generated from PCR products were cloned in pSPT18 vector and sequenced. DIG-labeled probes were conducted using a merchandized kit (Roche). Probes design was optimized in cooperation with the manufacturer (Shanghai Bogoo Biotechnology. Co. Ltd, China): forward primer: 5′-GGTTTTCTGCTCCTTGGTCC-3′; reverse primer: 5′-TTCTCTCTGGCATCGCTGAT-3′. Selection of the probe sequences (isoform primer forSAA1 and SAA2 mRNA) was based on the GenBank database. Experiments were carried out according to the manufacturer's recommendations. In brief, the slices were deparaffinized in xylene, digested by protease, fixed in neutral buffered formalin and denatured in the kit solution. Sections were prehybridized in mixture containing 5× SSC, pH 7.5/50% formamide for 1 h at 65^°^C in an oven. Following hybridization was performed at 37°C overnight (12– 16 h), then the slices were washed in washing buffer and counterstained with 4′, 6-diamidino-2-phenylindole (DAPI) in antifade solution, mounted with one drop of nonfluorescent oil and coverslipped. The slides were stored in dark before signal enumeration. The slides were examined by light microscopy (BX40; Olympus, Tokyo, Japan) with a fluorescent adapter, and digitally photographed to assess the intensities of positive immunostaining signal. To rule out false-positive or false-negative results, the positive and negative control tissues were processed together with the cancer tissues in the same staining batch but on different tissue slides.

### Statistical analysis

Statistical analyses were performed with SPSS 18.0 software (SPSS, Chicago, IL). Spearman's rank-correlation test was used to assess the association of SAA expression with clinicopathological characteristics. The cumulative survival times (overall survival, OS; recurrence-free survival, RFS) were calculated using the Kaplan–Meier method and analyzed with the log-rank test. Univariate and multivariate analyses were conducted based on the Cox proportional hazards regression model. All tests were two-sided, and a *p* value of less than 0.05 was considered statistically significant.
